# Risk factors of stunting and wasting in Somali pre-school age children: results from the 2019 Somalia micronutrient survey

**DOI:** 10.1186/s12889-021-12439-4

**Published:** 2022-02-09

**Authors:** William E. S. Donkor, Joshua Mbai, Fatmata Sesay, Sundus Ibrahim Ali, Bradley A. Woodruff, Shuaib Mohamoud Hussein, Kheyriya Mohamed Mohamud, Ahmed Muse, Warsame Said Mohamed, Abdullahi Muse Mohamoud, Farhan Mohamed Mohamud, Nicolai Petry, Melanie Galvin, Rita Wegmüller, Fabian Rohner, Yvonne Katambo, James P. Wirth

**Affiliations:** 1 GroundWork, Hintergasse 1, 7306, Fläsch, Switzerland; 2 Brandpro Ltd, Nairobi, Kenya; 3 UNICEF, Mogadishu, Somalia; 4 Department of Nutrition, Federal Ministry of Health, Mogadishu, Somalia; 5 Department of Nutrition, Ministry of Health, Hargeisa, Somaliland; 6 Department of Nutrition, Ministry of Health, Garowe, Puntland

**Keywords:** Stunting, Wasting, Micronutrients, iron, Children, Somalia, Risk factors, Malnutrition

## Abstract

**Background:**

Stunting and wasting in children less than 5 years of age are two key indicators of child malnutrition. Reducing their prevalence is a priority of the global public health community and for Somalia, a country suffering complex humanitarian emergencies such as drought, flooding, conflict and large-scale displacements.

**Methods:**

Data from the nationally representative cross-sectional Somalia Micronutrient Survey (SMS 2019) on 1947 children were analyzed to assess the prevalence and potential risk factors of stunting and wasting. Bivariate and multivariable analyses were conducted separately for children 0–5 months and 6–59 months, and population attributable fractions were calculated using adjusted risk ratios produced by Poisson regression models.

**Results:**

Among the 1947 children, the prevalence of stunting and wasting were 17.2% (95% CI: 15.0, 19.6) and 11.0% (95% CI: 9.3, 12.9), respectively. Among children 6–59 months of age, those residing in severely food insecure households had a higher risk of stunting (adjusted risk ratio [aRR] 1.47; CI: 1.12, 1.93) compared to those in food secure households. This risk of stunting was also higher in children with inflammation (aRR 1.75; CI: 1.35, 2.25) and iron deficiency (ID) (aRR 2.09; CI: 1.58, 2.80). For wasting, a dose-response relationship was found with household wealth, with the risk of wasting increasing significantly as the household wealth quintile decreased. On the other hand, the risk of wasting was lower in iron-deficient children (aRR 0.69; CI: 0.49, 0.98) than in iron-replete children. Among children 0–5 months of age no variables remained statistically significantly associated with stunting in the multivariable analysis. Wasting, however, was more common in children with recent diarrhea (aRR 3.51; CI: 1.68, 7.36).

**Conclusions:**

Nutritional status of children in Somalia may be improved by prevention of diarrhea and other infections and improvements in household food security.

**Supplementary Information:**

The online version contains supplementary material available at 10.1186/s12889-021-12439-4.

## Background

Stunting and wasting are two key nutrition indicators in children < 5 years of age. Globally, stunting affected 22.0% and wasting 6.7% of children under 5 years of age in 2020, and Africa has one of the highest stunting and wasting prevalence, only second to Asia [1].

Stunting is a result of suboptimal nutrition or long-term nutrition deprivation, which can occur in utero and during childhood [1, 2]. Short-term consequences of stunting include increased risk of infectious diseases [2], poor cognitive development [3], and increased morbidity [4]. The long-term consequences of stunting include reduced height and lean body mass in adulthood, along with decreased cognitive performance between 6 and 11 years of age and less educational attainment overall [5].

Wasting is due to inadequate nutrient intake and/or disease [6]. Wasted children have an acutely increased risk of death [1, 7] and therefore require urgent medical and nutrition treatment [7]. Wasting in young children often results in a weakened immunity and delayed physical development [1].

The prevalence of stunting and wasting in Africa currently stands at 41 and 27% respectfully. Regionally, the prevalence of stunting and wasting in Eastern Africa, Somalia included, has declined steadily from the year 2000 with the percentage of children stunted and wasted currently at 32.6 and 5.2% respectively. Stunting and wasting may co-exist [1], and the burden is highest in areas with ongoing conflicts [8]. In Somalia, humanitarian emergencies due to natural disasters and civil conflict put children below 5 years of age at particular risk of malnutrition. While monitoring of nutrition indicators is difficult under such circumstances [9], biannual cross-sectional surveys that include anthropometric measurements of children are often conducted in selected areas of Somalia [10]. Using pooled cross-sectional surveys from 2007 and 2010, researchers found that about 31 and 21% of children 6–59 months of age were stunted and wasted, respectively [6]. Based on this study, stunting and wasting prevalence in Somalia would be classified as “high” and “very high” according to WHO classifications [11]. More recent data from biannual cross-sectional surveys were available, but did not present national-level results.

The 2019 Somalia Micronutrient Survey (SMS) [12] was done to provide updated national estimates of stunting, wasting, and other nutritional deficiencies. Using anthropometric data collected from children and data on other household members and household characteristics, we aimed to identify the potential risk factors of stunting and wasting in children 0–5 months and 6–59 months of age. A detailed understanding of the risk factors of stunting and wasting in both age groups will serve as the basis for policy development and targeted interventions to address childhood undernutrition.

## Methods

### Study design

The 2019 SMS, a nationally-representative stratified cross-sectional household-based survey, was conducted between December 2018 and September 2019. Sampling was done from 6 strata: 1) Somaliland, 2) Puntland, 3) the Somalia states of Hirshabelle and Galmudug; 4) the Somalia states of Jubaland and South-West, 5) the Banaadir administrative region of Somalia, and 6) internally displaced persons (IDPs) settlements in all five aforementioned geographic strata combined.

The survey used a two-stage cluster sampling procedure. Enumeration areas (EAs) and IDP camps were primary sampling units which were selected using probability proportional to size. Within each selected primary sampling unit, the required number of households were selected using simple random sampling. In the first 5 geographic strata, 25 EAs were selected in each stratum after excluding EAs that were not accessible due to insecurity; EAs were categorized as either urban or rural. In the 6th stratum, 25 IDP camps were selected. In each EA and IDP camp, 16 households were randomly selected resulting in a total sample of 2400 households. Further details of the selection procedure can be found elsewhere [12].

Within participating households, all children 0–59 months of age were eligible to be recruited into the survey, questionnaires in the child questionnaire were answered by the selected child’s mother or caretaker. Non-pregnant women were recruited from a randomly selected one-half subsample.

### Questionnaire data

Data on household characteristics were collected from the household head or knowledgeable adult household member. The household interview collected information on the household’s dwelling, durable goods ownership, water source, and sanitation facility using standard questions widely used in health and nutrition assessment surveys worldwide. Durable goods and dwelling characteristics were used to calculate a household wealth index using the standard methods [13, 14]. Household-level sanitation facilities and water source were respectively classified as adequate/inadequate or safe/unsafe based on WHO/UNICEF guidelines [15]. To estimate sanitation status at the community-level, the proportion of households in each cluster with inadequate sanitation facilities was calculated and categorized into sub-groups of 0–19%, 20–39%, 40–59%, 60–79%, and 80–100%. In addition, household food security was assessed using the Household Food Insecurity Access Scale (HFIAS) questionnaire module. The final scale was categorized into four categories: food secure, mild food insecurity, moderate food insecurity, and severe food insecurity [16].

The child questionnaire collected data on age, sex, recent morbidity, and consumption of vitamin and mineral supplements. Child age was calculated by subtracting the child’s date of birth from the date of the interview. As much of the population in Somalia was not calendar literate, a local event calendar for the past 5 years was developed to facilitate the approximation of the child’s birth date. The child questionnaire included standard infant and young child feeding questions [17] that were administered to children 0–23 months of age. Questionnaire data was also collected from women, however, as women were only recruited from ½ of all households, maternal-level data was not included in this analysis. The Open Data Kit (ODK) software was used for direct electronic questionnaire data entry.

### Anthropometric assessments

Anthropometrists were trained in standard anthropometric techniques [18]. Performance during a standardization exercise, the results of a post-test, and observations from trainers were used to select the best performing team members. All survey procedures were practiced in a field test under close supervision prior to the start of data collection.

During the survey field work, the weight and height or length of each child was measured according to standard procedures [19]. Child weight was measured using an automatically taring bathroom scale (SECA®, Hamburg, Germany); all scales were calibrated each morning before the start of data collection. The standard wooden height board (UNICEF item number S0114540, Copenhagen, Denmark) was used to measure the child height or length. Length/height and weight measurements were taken twice by the same measurer and both values recorded. The second length/height measurement was taken after the child was removed and replaced on the height board to account for incorrect positioning during the first height measurement. For weight measurements, children that could not stand were directly weighed on the scale, and the scale’s tare function was used for children that could not stand. For the second measurement, children were either re-weighed directly or the tare process was repeated.

### Blood biomarkers

Capillary blood was collected from all children 6–59 months; no blood was taken from children younger than 6 months of age to prevent potential injury. Blood was collected from the heel in children 6–11 months of age and from the finger in children 12–59 months of age. After sterilization with an alcohol pad, the puncture site was wiped dry and punctured with a “contact activated” high-flow blade lancet (Becton Dickinson, Franklin Lakes, NJ, USA). After wiping the first drop of blood away, the second and third drop of blood were used to measure hemoglobin concentration and recent or current malaria infection, followed by the collection of 300–400 μl of capillary blood into a silica-coated blood collection tube (Sarstedt, Microvette® 300 Z, Nümbrecht, Germany).

Following on-site measurements of haemoglobin and malaria parasitemia, the labeled microtainers were placed in a cool box at 2–8 °C in the dark for transport in the evening of the same day to one of four state laboratories. Samples were centrifuged at 3000 rpm for 7 min to separate the serum, which was then aliquoted into labeled cryovials. Aliquots were stored at − 20 °C and later shipped on dry ice for analyses. Serum samples were analyzed for retinol-binding protein (RBP), ferritin, C-reactive protein (CRP), and alpha 1-acid glycoprotein (AGP) at the VitMin-Lab (Wilstaett, Germany) using an ELISA method.

### Data management and statistical analysis

Anthropometry, anemia and malaria data were collected on paper forms, and subsequently entered into ODK on the same day. Data analysis was done using Stata/IC version 14.2. All analyses of questionnaire data were conducted using sampling weights to account for the unequal probability of selection in the six strata.

Z-scores were calculated based on WHO’s 2006 Growths Standards [20], and children with height-for-age z-scores (HAZ) and weight-for-height z-scores (WHZ) < − 2.0 standard deviations were classified as stunted and wasted, respectively [20]. Prior to data analysis, the quality of anthropometric measurements was examined for all teams. As a result of this examination, child height measurements taken by all teams working in Galmudug State were excluded, and length, height, and weight measurements taken by one team in South-West State and one team in Jubaland State were excluded [12]. Further details about anthropometric data quality are presented elsewhere [12].

Any inflammation was defined as concentrations of CRP > 5 mg/L and/or concentrations of AGP > 1 g/L [21]. After adjusting serum ferritin and RBP concentrations for inflammation using the *Biomarkers Reflecting Inflammation and Nutritional Determinants of Anemia* (BRINDA) approach, iron deficiency (ID) was defined as serum ferritin concentrations < 12 μg/L [22] and vitamin A deficiency was defined as RBP concentrations < 0.7 μmol/L [23].

For categorical variables, proportions were calculated to derive the prevalence. All measures of precision, including 95% confidence limits and chi square *p* values for differences in subgroup prevalence, were calculated accounting for the complex cluster and stratified sampling used by the SMS 2019.

For this analysis, we identified potential risk factors from the recent WHO conceptual framework for stunting [24] and other potential risk factors of wasting identified from a review of the literature. Bivariate and multivariable analyses were conducted separately for children 0–5 months and 6–59 months to identify potential risk factors of stunting and wasting. Separate analyses for these age groups were done to account for the fact that blood biomarkers were only available in children 6–59 months of age. For all analyses, significance was accepted at *P < 0.05.*

Variables with chi-square *p*-values < 0.1 during categorical bivariate analyses were included in the four subsequent multivariable models. When applicable during the bivariate analysis, a nonparametric test for trend was conducted using Stata’s *nptrend* command [25] to identify dose-response relationships. Following tests of collinearity (i.e., variance inflation factor), multivariable Poisson regression models were run using backwards elimination until only variables with statistically significant associations were remaining. To account for age differences, age in months was included in all regression models.

Following the multivariable analyses, the population attributable fraction (PAF) was calculated for all statistically significant potential risk factors. PAF was calculated using the equation $pd\ \left(\frac{aRR-1}{aRR}\right)$ using the adjusted risk ratios (aRRs) produced by the Poisson regressions and the proportion of cases with the potential risk factor of interest (pd) [26].

## Results

### Description of survey sample

Table 1 shows the demographic characteristics of children included in the survey sample. Of the 1947 children recruited into the survey, approximately 20% are found in each 1-year age group between 12 and 59 months of age, however, children between 0 and 11 months accounted for only 15.8% of all children surveyed. The number of males and females were evenly distributed, and approximately half of the children resided in urban areas, one-third in rural areas, and 15% in IDP settlements.

**Table 1:** Description of sampled children (0–59 months), Somalia 2019

Characteristic	Survey Sample		
	n	% ^**a**^	(95% CI) ^**b**^
Age Group (in months)			
0–5	136	7.3	(5.8, 9.1)
6–11	165	8.5	(7.3, 9.8)
12–23	386	19.7	(17.7, 21.8)
24–35	434	22.6	(20.8, 24.5)
36–47	436	22.3	(20.3, 24.3)
48–59	390	19.8	(17.9, 21.8)
Sex			
Male	979	50.9	(48.5, 53.4)
Female	968	49.1	(46.6, 51.5)
Residence			
Rural	626	31.9	(23.9, 41.0)
Urban	1042	53.2	(44.4, 61.8)
IDP	279	15.0	(12.7, 17.6)
ALL CHILDREN	1947	100.0%	–

Note: The n’s are un-weighted numbers of subjects in each subgroup; the sum of subgroups may not equal the total because of missing data

^a^Percentages are un-weighted and do not account for unequal probability of selection

^b^*CI* Confidence interval, calculated taking into account the complex sampling design

### Distribution of stunting prevalence in children 0–59 months

**Fig. 1:**
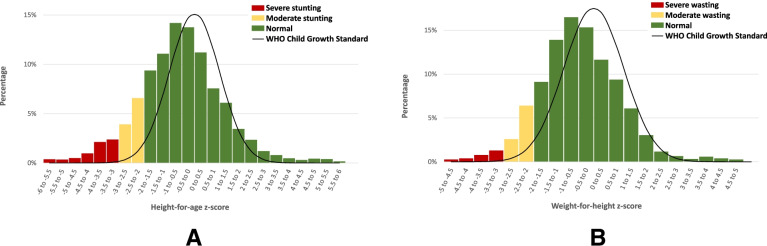
Distribution of height-for-age z-scores (**A**) and weight-for-height z-scores (**B**), children 0–59 months of age, Somalia 2019

In children 0–59 months of age, the stunting prevalence was 17.2% (95% CI: 15.0, 19.6), and the weighted mean and standard deviation of HAZ was − 0.50 and 1.73, respectively. The prevalence of stunting is considered a medium public health problem according to WHO’s criteria [27]. As shown in panel A of Fig. 1, the HAZ distribution is shifted to the left of the WHO Growth Standards, illustrating that children in Somalia are slightly shorter than children from healthy reference populations [20]. Moderate and severe stunting were found in 10.5% (95% CI: 8.9, 12.3) and 6.7% (95% CI: 5.4,8.3) of children in this age group, respectively. The prevalence of stunting was higher among male children (19.3%; 95% CI: 16.1, 23.0) than female children (15.0%; 95% CI: 12.3, 18.1), although this difference was not strictly statistically significant (*p* = 0.055).

### Distribution of wasting prevalence in children 0–59 months

In children 0–59 months of age, the prevalence of wasting was 11.0% (95% CI: 9.3, 12.9), denoting a serious public health problem according to the WHO thresholds [27]. The mean and standard deviation of WHZ were − 0.48 and 1.35, respectively. As shown in panel B of Fig. 1, the WHZ is shifted to the left of the WHO Child Growth Standards [20]. Severe wasting was found in fewer children (2.3%; 95% CI: 1.7, 3.2) than moderate wasting (8.6%; 95% CI: 7.1, 10.4). The prevalence of wasting was higher among male children (12.2%; 95% CI: 9.8, 15.1) than female children (9.7%; 95% CI: 7.7, 12.1), but the difference was not statistically significant (*p* = 0.12).

### Bivariate associations between stunting and various potential risk factors in children 0–5 months and 6–59 months of age

The prevalence of stunting in children 0–5 and 6–59 months of age by various potential risk factors is provided in Supplementary Table S1.

The prevalence of stunting in children 0–5 months of age was 10.9% (95% CI: 6.1, 18.7). Only one potential risk factor showed a statistically significant association with stunting in the bivariate analysis. The prevalence of stunting was unexpectedly higher in children residing in households with adequate sanitation facilities compared to children in households with inadequate sanitation facilities (17.1% vs 2.8%, respectively; *p* < 0.005).

The prevalence of stunting in children 6–59 months of age was 17.8% (95% CI: 15.5, 20.3). Stunting was significantly associated with age (*p <* 0.001), with the highest prevalence found among children 24–35 months of age (23%) and the lowest prevalence (11.5%) found in both the youngest and the oldest age groups. Stunting prevalence was higher in IDP settlements compared to rural and urban settlements (28.4, 13.8, and 16.5%, respectively; *p* < 0.01). In addition, stunting prevalence was substantially higher in South-West State (38.9%; *p* < 0.001) than in other states; the next highest prevalence of stunting was 25.6% in Banaadir. The prevalence of stunting showed a progressive decline with increasing household wealth (*p <* 0.005), from 33.9% in the poorest quintile to 11.5% in the richest quintile. The nonparametric test for trend found a statistically significant (*p* < 0.001) decrease in stunting prevalence by increasing household wealth. The stunting prevalence among children with inflammation was almost double the prevalence in children with no inflammation (26.1% vs 13.8%; *p* < 0.001). Children in households in which soap was available had a lower prevalence of stunting than children in households in which soap was not available (14.6% vs. 22.7%; *p* < 0.01). The stunting prevalence showed a progressive increase with increasing food insecurity, from 13.5% in children in food secure households to 21.8% in children in households with severe food insecurity (*p* < 0.01). A statistically significant increase (*p* < 0.001) in the prevalence of stunting as by food insecurity group was observed with the nonparametric test for trend.

### Multivariable analysis - risk factors of stunting in children 0–5 months and 6–59 months of age

As a first step in the model building process, collinearity between the potential risk factors was explored by calculating the variance inflation factor for the preliminary models. Variance inflation factor results were consistently < 2 indicating that there was no collinearity.

Table 2 presents the final parsimonious multivariable models for stunting in children 0–5 and 6–59 months of age. In children 0–5 months, no variable remained statistically significant for stunting in the model. In contrast, in older children, several variables remain statistically significantly associated with stunting. Iron deficiency accounted for approximately one-third of stunting in this age group, while inflammation and severe household food insecurity accounted for less than 20%. Unlike severe household food insecurity, mild and moderate food insecurity were not statistically significantly associated with stunting.

**Table 2:** Multivariable analysis: Adjusted relative risk and population attributable fraction of stunting and wasting in children 0–5 months and 6–59 months, Somalia 2019

Characteristic	Category	***Adjusted relative risk***	***95% CI***	Population attributable fraction ^**b**^
***0–5 months***^***a***^				
No variables significant				
***6–59 months (n = 1179)***^***a***^				
Inflammation ^c^	Yes	1.75	(1.35, 2.25)	16.9%
	No	referent		
Iron status ^d^	Deficient	2.09	(1.58, 2.80)	34.6%
	Sufficient	referent		
Household food security status	Severe food insecurity	1.47	(1.12, 1.93)	17.1%
	Moderate food insecurity	1.33	(0.88, 2.03)	2.3%
	Mild food insecurity	0.86	(0.44, 1.69)	−0.7%
	Food secure	referent		
***0–5 months (n = 102)***^***a***^				
Diarrhea in past 2 weeks	Yes	3.51	(1.68, 7.36)	28.0%
	No	referent		
Exclusive breastfeeding ^e^	Yes	0.00	(0.00, 0.00)	–
	No	referent		
***6–59 months (n = 1178)***^***a***^				
Iron status ^d^	Deficient	0.69	(0.49, 0.98)	−18.2%
	Sufficient	referent		
Household wealth quintile	Lowest	2.24	(1.02, 4.87)	4.5%
	Second	2.21	(1.27, 3.82)	10.7%
	Middle	1.95	(1.23, 3.10)	14.5%
	Fourth	1.33	(0.81, 2.21)	5.7%
	Highest	Referent	–	

^a^Age in months as a continuous variable included in all models. ^b^Population attributable fraction calculated using adjusted relative risk from Poisson regression. ^c^Inflammation defined as elevated CRP (> 5 mg/L) and/or elevated AGP (> 1 g(L). ^d^Iron deficiency defined as inflammation-adjusted (using BRINDA approach) serum ferritin < 12 μg/L. ^e^Relative risk of wasting for exclusive breastfeeding was nearly zero, with relative risk and associated confidence intervals represented in scientific notation (2.92e-08; 95% CI: 1.51e-08, 5.64e-08)

### Bivariate associations between wasting and various potential risk factors in children 0–5 months and 6–59 months of age

The wasting prevalence for various potential risk factors in children 0–5 and 6–59 months of age by is provided in Supplementary Table S2.

The prevalence of wasting in children 0–5 months of age was 17.2% (95% CI: 9.2, 30.0). Of the various potential risk factors explored in this age group, only diarrhea and exclusive breastfeeding were significantly associated with wasting. The wasting prevalence was significantly higher among children who had diarrhea in the past 2 weeks compared to those without diarrhea (53.3% vs. 12.0%; respectively; *p <* 0.001). Wasting prevalence was also lower among exclusively-breastfed children compared to those not exclusively breastfed (0.0% vs 21.7%, respectively; *p* = 0.093).

The prevalence of wasting in children 6–59 months of age was 10.5% (95% CI: 8.9, 12.4). Wasting was significantly associated with age (*p* < 0.05), children 48–59 months of age had the highest wasting prevalence (15.0%). State of residence (*p <* 0.01) was also significantly associated with wasting. The highest prevalence, found in Jubaland (15.9%), constitutes a severe public health problem according to WHO classification standards [27]. Household wealth was significantly associated with wasting, with a suggestion of a decline in the prevalence with increasing household wealth. Although the wasting prevalence did not decrease for all household wealth groups (i.e., wasting increased from 11.7% in the poorest group to 16.4% in second group), the nonparametric test for trend showed a statistically significant (*p* < 0.01) decrease in wasting as wealth group increased. Children with LRI in the past 2 weeks had significantly higher levels of wasting compared to children with no LRI (16.4% vs. 10.1%; *p <* 0.05).

### Multivariable analysis - risk factors of wasting in children 0–5 months and 6–59 months of age

Prior to backwards elimination, variance inflation factor calculations were conducted for the preliminary models that contained all variables associated with wasting at *p* < 0.1 during the bivariate analyses. Variance inflation factor results were consistently < 2 for all risk factors in all models, showing no collinearity in the preliminary models.

In children 0–5 months of age, two variables were strongly associated with wasting: recent diarrhea and exclusive breastfeeding. Children with a history of recent diarrhea were more than 3 times as likely to be wasted as children without such a history, and approximately 30% of wasting in this age group was attributable to recent diarrhea. Children 0–5 months of age who were exclusively breastfed had essentially no risk of being wasted. Due to the lack of variability between wasting and exclusive breastfeeding, no population attributable fraction could be calculated.

In older children, iron deficiency was protective against wasting. As a result, the population attributable fraction is negative. Household wealth showed a dose-response relationship with wasting in this age group; however, the lowest wealth quintiles contributed less to wasting than did the middle wealth quintiles.

## Discussion

### Stunting in children 0–5 and 6–59 months of age

As expected, our study found that factors associated with stunting were different in children 0 to 5 months of age and children 6 to 59 months of age. The lack of significantly associated variables with stunting in children 0 to 5 months of age could be due to the small sample size in our study. This survey’s sampling scheme selected children 0 to 5 months of age in proportion to their representation in the larger population of children less than 5 years of age. As a result, they represent about 10% of our total survey sample of children. Additionally, stunting in this age group may stem predominantly from antenatal factors which were not measured in this survey. In an analysis of data from 137 developing countries, Danaei et al. concluded that fetal growth restriction was the main risk factor of stunting [28]. Few children surveyed in the SMS 2019 had been weighed at birth, and data on estimated birth size — a proxy for being born small for gestational age — was not collected. As our study did not collect blood samples from children 0–5 months of age, we could not examine various biomarkers, such as inflammation, which has been associated with children < 6 months of age in other countries [29, 30] and was associated with stunting in older children in our study.

In children 6–59 months of age, inflammation, household food security status, and ID were the only variables statistically significantly associated with stunting in the final regression model. Inflammation has previously been identified as a potential risk factor for growth faltering in a case control study of children 22–28 months of age from northern Tanzania, where elevated AGP levels were associated with lower HAZ [31]. In addition, a case-control study nested in a cohort study of Zimbabwean children [30] used archived plasma specimens to demonstrate higher CRP and AGP concentrations in the first year of life among children who were stunted at 18 months of age. This same study also found inverse associations between CRP and AGP and insulin-like growth factor 1 (IGF-1), a hormone that stimulates bone, muscle, and organ growth [30]. Generally, inflammation has been repeatedly associated with increased cytokine production, which in turn suppresses bone tissue formation and increases cellular destruction [32]. In other studies, inflammation has been attributed to enteropathy and gut malfunction caused by exposure to poor sanitation and hygiene conditions [33]. In contrast, our study found no associations in the multivariable models between stunting or wasting and the sanitation- or hygiene-related variables examined. Ancillary analyses also found no association between a child’s inflammation status and his/her household’s sanitation level (i.e., adequate vs. inadequate) or the community-level prevalence of open defecation (data not shown). The association between inflammation and community-level open defectation may be confounded by the fact that our study did not examine the population density in each surveyed community. Using data from Bangladesh, Hathi et al. [34] have found that open defecation has stronger associations with child health in high-density communities.

In this study, household food insecurity was measured using the HFIAS, and severe household food insecurity accounted for a substantial proportion of stunting in children 6–59 months of age. Psaki et al. [35] examined associations between HFIAS and child HAZ and found a significant association between higher levels of household food insecurity and lower HAZ.

Although we identified ID as a potential risk factor for stunting, the mechanism by which ID contributes to stunting is not clear. It is possible that ID may be a proxy for the lack of dietary diversity or diet poor in other micronutrients. A cross-sectional study in Indonesia found that household-level consumption of iron-rich foods, such as fish, meat, and poultry, was associated with lower prevalence of stunting among children < 5 years of age [36].

### Wasting in children 0–5 and 6–59 months of age

As with stunting, the factors associated with wasting in children 0–5 months and 6–59 months of age were different. In children 0–5 months of age, the association in younger children between recent diarrhea and wasting is unsurprising. A recent systematic review of studies examining the risk factors of wasting in sub-Saharan Africa found that diarrhea was one of the most consistent factors associated wasting [37]. Diarrhea can *directly* affect nutritional status via the loss of nutrients and fluids and by reducing food intake and nutrient absorption [38]. In addition, wasting can *indirectly* lead to diarrhea by predisposing a child to infections by compromising his/her immune function and increasing intestinal permeability [38]. Furthermore, concurrent wasting and diarrhea have also been shown to increase the incidence of mortality in children < 5 years of age [39, 40]. Our finding that no exclusively breast-fed child had recent diarrhea is consistent with, albeit somewhat more extreme than, the results of a recent meta-analysis [41]. Our multivariable modelling demonstrated independent effects of both recent diarrhea and exclusive breastfeeding on wasting; however, exclusive breastfeeding has been associated with lower prevalence of recent diarrhea and other diseases [42]. As a result, exclusive breastfeeding decreases wasting by more than one causative pathway.

Our study seems to identify an inverse association between ID and wasting. This finding is perplexing, as a recent systematic review and meta-analysis that examined the influence of low-dose iron supplementation on child growth found no association between iron status and WHZ, wasting, HAZ, or stunting [43]. Moreover, one could assume, as with stunting, that ID would denote a non-diverse diet, that could potentially make a child more likely to be wasted. ID has been shown to be protective against malaria [44], which could contribute to growth faltering [45]. However, this explanation does not relate to our findings as the 2019 SMS identified malaria in very few children (*n* = 9), none of which were wasted. Another hypothetical explanation could be that non-wasted children are fed more complementary foods containing iron-absorption inhibitors (e.g. whole grain porridge) [46]; thus improving growth whilst decreasing iron status. However, exploratory analysis of 24-h food frequency data — collected as part of our study from children 6–23 months of age — did not reveal any significant associations between wasting and consumption of grains. Alternatively, the protective effect of ID we found may suggest that there is a confounding variable not included in our analysis that is producing this spurious association.

The substantially elevated risk of wasting among the poorest children is consistent with the findings of other studies: An analysis of data from Ghana’s DHS demonstrated an association between wasting and household wealth, with children in households within the poorest wealth quintile being more likely to be wasted [47]. Furthermore, a systematic review of studies on children under 5 years in sub-Saharan Africa [48] found that wasting was associated with low household wealth in Ghana, Nigeria, Ethiopia, and Kenya. Several studies across sub-Saharan Africa [49–51] report that higher household income and food security and greater healthcare utilization lead to improved child nutritional outcomes.

### Limitations

Our analysis did not contain certain potential key risk factors for stunting and wasting. As shown in other studies, there are many variables that influence child growth and development [52]. For example, child birthweight or estimated size at birth could have been collected as indicators of growth restriction in utero. However, few children included in the 2019 SMS were weighed at birth. Further, maternal age, education, and profession have been associated with child stunting throughout sub-Saharan Africa region [37], but maternal risk factors of stunting and wasting could not be included in our analyses because non-pregnant women were only selected from a subsample of enrolled households. As a result, maternal information was not available for one-half of enrolled children.

Our study also did not collect stool samples from children in order to assess the presence of enteropathogens. While stool samples are rarely collected as part of national surveys, enteropathogens have been linked with child growth in other studies [53, 54], and accounting for these variables would have enhanced our analyses. Dietary intake was also not included, which prevented the analysis of the associations between growth and protein intake, an association that has been observed in other studies [55]. Another limitation of our analyses is that no blood samples were taken from children 0–5 months of age, and thus, no associations between stunting and wasting and biomarker indicators, such as anemia, iron deficiency, and inflammation, could be assessed in this group. In addition, the sample size of children 0–5 months of age was quite small, which likely affected our ability to identify significant risk factors for stunting and wasting in this age group.

Although anemia was not a significant risk factor of stunting or wasting, the use of capillary blood samples to measure hemoglobin concentration can be seen as a limitation to our study. When comparing the hemoglobin concentrations in capillary and venous blood samples in the same individuals from seven countries, Rappaport et al. [5] found consistently lower hemoglobin concentrations in the capillary samples. The authors posited that this may be due to increased levels of interstitial fluid — and conversely less hemoglobin — in capillary samples incurred through improper blood collection techniques, such as excessive squeezing of a child’s finger during blood collection. While the phlebotomists were instructed to not to squeeze or “milk” a child’s finger, the collection of venous blood samples would have plausibly increased the accuracy and precision of the hemolgobin measurements.

The standard deviations of HAZ and WHZ values are higher than those recommended by the SMART guidelines [56], which may indicate suboptimal quality of anthropometric measurements. On the other hand, these standard deviations are similar to those from DHS surveys in other African countries [57]. We observed substantial age heaping [58] which may have increased the variability of HAZ values. Despite the use of local events calendars, widespread calendar illiteracy and lack of child health cards resulted in relatively poor accuracy in the estimates of child dates of birth.

Regarding studies comparing haemoglobin results in the same individuals, a recent study by Rappaport et al. [59] combined data from 11 separate studies from seven countries with samples from children, men, non-pregnant women and pregnant women. In addition to using previously unpublished data, Rappaport et al. [59] included results from previously-published studies that compared haemoglobin measurement methods [60–65].

## Conclusions

The risk factors associated with stunting and wasting differ in children 0–5 months and 6–59 months of age, suggesting that differing interventions are required to improve child growth at various growth stages. A notable proportion of malnutrition was attributable to infection-related factors (i.e., inflammation and diarrhea), suggesting that interventions that limit a child’s exposure to infection-causing pathogens would improve child growth. The dose-response associations between child growth and household food insecurity and household wealth suggest that household-level social protection interventions would have that ancillary benefit of reducing the prevalence of stunting and wasting.

## Supplementary Information

**Additional file 1: Supplementary Table S1.** Prevalence of stunting by potential risk factors in children 0 - 5 months and children 6-59 months of age, Somalia 2019. **Supplementary Table S2.** Prevalence of wasting by potential risk factors in children 0 - 5 months and children 6-59 months of age, Somalia 2019.

## Data Availability

The datasets used and/or analysed during the current study are available from the corresponding author on reasonable request.

## Abbreviations

HFIAS: Household Food Insecurity and Access Scale
SMS: Somalia Micronutrient Survey
WHO: World Health Organization
ID: Iron deficiency
