# Supplementary material for: Risk factors of stunting and wasting in Somali pre-school age children: results from the 2019 Somalia micronutrient survey
Source: BMC Public Health. 2022 Feb 9;22:264. doi: 10.1186/s12889-021-12439-4 (PMC8827289; doi:10.1186/s12889-021-12439-4)
Supplement: Supplementary file 1 — Additional file 1: Supplementary Table S1. Prevalence of stunting by potential risk factors in children 0 - 5 months and children 6-59 months of age, Somalia 2019. Supplementary Table S2. Prevalence of wasting by potential risk factors in children 0 - 5 months and children 6-59 months of age, Somalia 2019. [file 12889_2021_12439_MOESM1_ESM.docx]

**Supplementary Table S1. Prevalence of stunting by potential risk factors in children 0 - 5 months and children 6-59 months of age, Somalia 2019**

| **Characteristic** | **Any Stunting, 0-5 months (N=104)** | | | | **Any Stunting, 6-59 months (N=1,443)** | | | |  |
| --- | --- | --- | --- | --- | --- | --- | --- | --- | --- |
|  | **n** | **% ^a^,^d^** | **(95% CI) ^b^** | **P-value ^c^** | **n** | **% ^a^,^d^** | **(95% CI) ^b^** | **P-value ^c^** |  |
| Age Group (in months) |  |  |  |  |  |  |  |  |  |
| 0-5 | 11 | 10.9 | (6.1, 18.7) | - | - | - | - |  |  |
| 6-11 | - | - | - |  | 15 | 11.5 | (7.0, 18.5) | 0.001 |  |
| 12-23 | - | - | - |  | 62 | 20.2 | (15.9, 25.3) |  |  |
| 24-35 | - | - | - |  | 76 | 23.0 | (18.0, 29.0) |  |  |
| 36-47 | - | - | - |  | 59 | 18.8 | (14.4, 24.2) |  |  |
| 48-59 | - | - | - |  | 42 | 11.5 | (8.9, 14.7) |  |  |
| Sex |  |  |  |  |  |  |  |  |  |
| Male | 6 | 13.6 | (6.3, 27.0) | 0.441 | 144 | 19.7 | (16.4, 23.5) | 0.071 |  |
| Female | 5 | 8.5 | (3.2, 20.5) |  | 110 | 15.5 | (12.7, 18.8) |  |  |
| Residence |  |  |  |  |  |  |  |  |  |
| Rural | 3 | 10.2 | (2.9, 30.0) | 0.483 | 55 | 13.8 | (10.6, 17.9) | 0.003 |  |
| Urban | 8 | 12.9 | (6.9, 22.8) |  | 140 | 16.5 | (13.6, 19.8) |  |  |
| IDP |  |  |  |  | 59 | 28.4 | (19.9, 38.6) |  |  |
| State |  |  |  |  |  |  |  |  |  |
| Somaliland | 4 | 9.9 | (4.3, 21.1) | 0.250 | 64 | 12.6 | (9.6, 16.3) | 0.001 |  |
| Puntland | 3 | 9.5 | (3.2, 25.0) |  | 42 | 14.4 | (10.2, 19.9) |  |  |
| Hirshabelle | 0 | 0 | - |  | 26 | 13.9 | (9.5, 19.9) |  |  |
| South-West | - | - | - |  | 24 | 38.9 | (24.9, 55.0) |  |  |
| Jubaland | 1 | 20.0 | (2.4, 71.4) |  | 8 | 20.5 | (13.3, 30.3) |  |  |
| Banaadir | 3 | 34.7 | (9.1, 73.9) |  | 90 | 25.6 | (20.0, 32.2) |  |  |
| Wealth Quintile |  |  |  |  |  |  |  |  |  |
| Poorest | 0 | 0.0 | - | 0.849 | 24 | 33.9 | (22.1, 48.3) | 0.002 |  |
| Second | 2 | 18.9 | (4.7, 52.3) |  | 29 | 21.6 | (14.1, 31.5) |  |  |
| Middle | 2 | 7.4 | (1.6, 28.5) |  | 80 | 21.0 | (15.9, 27.1) |  |  |
| Fourth | 4 | 10.0 | (4.0, 23.2) |  | 72 | 14.6 | (11.2, 18.7) |  |  |
| Richest | 3 | 11.7 | (4.7, 26.3) |  | 46 | 11.5 | (7.3, 17.6) |  |  |
| Exclusive breastfeeding |  |  |  |  |  |  |  |  |  |
| No | 7 | 8.8 | (4.2, 17.3) | 0.153 | - | - | - |  |  |
| Yes | 4 | 19.4 | (7.6, 41.2) |  | - | - | - |  |  |
| Ever breastfed |  |  |  |  |  |  |  |  |  |
| No | 2 | 21.8 | (6.2, 54.0) | 0.223 | - | - | - |  |  |
| Yes | 9 | 9.1 | (4.5, 17.4) |  | - | - | - |  |  |
| Diarrhea in past 2 weeks |  |  |  |  |  |  |  |  |  |
| No | 11 | 12.3 | (6.9, 21.0) | 0.200 | 211 | 17.1 | (14.6, 20.0) | 0.271 |  |
| Yes | 0 | 0.0 | -- |  | 43 | 21.2 | (15.3, 28.5) |  |  |
| LRI in past 2 weeks |  |  |  |  |  |  |  |  |  |
| No | 11 | 11.4 | (6.4, 19.7) | 0.443 | 239 | 18.0 | (15.7, 20.5) | 0.246 |  |
| Yes | 0 | 0.0 |  |  | 15 | 13.4 | (7.8, 22.0) |  |  |
| Inflammation |  |  |  |  |  |  |  |  |  |
| No | - | - | - |  | 126 | 13.8 | (11.4, 16.5) | 0.001 |  |
| Yes | - | - | - |  | 76 | 26.1 | (21.1, 31.7) |  |  |
| Iron status |  |  |  |  |  |  |  |  |  |
| Sufficient | - | - | - |  | 67 | 11.0 | (8.7, 13.9) | 0.001 |  |
| Deficient | - | - | - |  | 135 | 23.2 | (19.2, 27.7) |  |  |
| Vitamin A status |  |  |  |  |  |  |  |  |  |
| Sufficient | - | - | - |  | 121 | 15.6 | (12.7, 19.1) | 0.158 |  |
| Deficient | - | - | - |  | 81 | 19.4 | (15.5, 23.9) |  |  |
| Household sanitation ^f^ |  |  |  |  |  |  |  |  |  |
| Inadequate | 2 | 2.8 | (0.7, 10.1) | 0.005 | 148 | 19.7 | (16.2, 23.6) | 0.086 |  |
| Adequate | 9 | 17.1 | (9.1, 29.9) |  | 103 | 15.0 | (11.8, 18.8) |  |  |
| Safe drinking water |  |  |  |  |  |  |  |  |  |
| Unsafe | 1 | 3.4 | (0.4, 23.0) | 0.193 | 43 | 16.4 | (11.5, 22.8) | 0.673 |  |
| Safe | 10 | 12.4 | (6.7, 21.8) |  | 208 | 17.8 | (15.2, 20.7) |  |  |
| Any Soap |  |  |  |  |  |  |  |  |  |
| No | 1 | 2.6 | (0.3, 17.4) | 0.074 | 100 | 22.7 | (17.8, 28.6) | 0.006 |  |
| Yes | 10 | 13.5 | (7.6, 23.0) |  | 151 | 14.6 | (12.2, 17.4) |  |  |
| Household food security |  |  |  |  |  |  |  |  |  |
| Food secure | 5 | 13.4 | (6.1, 26.9) | 0.510 | 88 | 13.5 | (10.6, 16.9) | 0.008 |  |
| Mild insecurity | 1 | 26.6 | (3.2, 79.8) |  | 10 | 14.5 | (7.7, 25.8) |  |  |
| Moderate insecurity | 1 | 8.6 | (1.1, 44.3) |  | 29 | 17.8 | (12.5, 24.7) |  |  |
| Severe insecurity | 4 | 7.1 | (2.5, 18.7) |  | 24 | 21.8 | (17.8, 26.5) |  |  |
| Percent inadequate sanitation in cluster |  |  |  |  |  |  |  |  |  |
| 0-19% | 2 | 8.6 | (3.0, 22.2) | 0.184 | 29 | 14.5 | (9.5, 21.7) | 0.408 |  |
| 20-39% | 3 | 11.7 | (4.0, 30.0) |  | 55 | 16.3 | (12.5, 21.0) |  |  |
| 40-59% | 6 | 22.6 | (11.1, 40.7) |  | 62 | 16.6 | (12.5, 21.7) |  |  |
| 60-79% | 0 | 0.0 | -- |  | 44 | 16.3 | (10.0, 25.2) |  |  |
| 80-100% | 0 | 0.0 | -- |  | 61 | 22.5 | (16.2, 30.2) |  |  |
| **ALL CHILDREN** | **11** | **10.9** | **(6.1, 18.7)** |  | **254** | **17.6** | **(15.4, 20.4)** |  |  |
| Note: The n’s are un-weighted numerators for each subgroup; subgroups that do not sum to the total have missing data.  ^a^ Percentages weighted for unequal probability of selection  ^b^ CI=confidence interval, calculated taking into account the complex sampling design  ^c^ P-value <0.05 indicates that at least one subgroup is statistically significantly different from the other subgroups.  ^d^ Any stunting is defined as HAZ < -2 calculated using the WHO Child Growth Standards  ^f^ Composite variable of toilet type and if toilet facilities are shared with non-household members; Adequate Sanitation = flush or pour flush toilet or pit latrine with slab not shared with another household. Inadequate sanitation= open pit, bucket latrine, hanging toilet/latrine, no facility, bush, field | | | | | | | | | |

**Supplementary Table S2. Prevalence of wasting by potential risk factors in children 0 - 5 months and children 6-59 months of age, Somalia 2019**

| **Characteristic** | **Any Wasting, 0-5 months (N=102)** | | | | **Any Wasting, 6-59 months (N=1,445)** | | | |  |
| --- | --- | --- | --- | --- | --- | --- | --- | --- | --- |
|  | **n** | **% ^a^,^d^** | **(95% CI) ^b^** | **P-value ^c^** | **n** | **% ^a^,^d^** | **(95% CI) ^b^** | **P-value ^c^** |  |
| Age Group (in months) |  |  |  |  |  |  |  |  |  |
| 0-5 | 19 | 17.2 | (9.2, 30.0) | - | - | - | - |  |  |
| 6-11 | - | - | - |  | 16 | 8.2 | (5.1, 12.9) | 0.027 |  |
| 12-23 | - | - | - |  | 35 | 10.2 | (7.3, 14.3) |  |  |
| 24-35 | - | - | - |  | 25 | 7.4 | (5.0, 10.9) |  |  |
| 36-47 | - | - | - |  | 33 | 10.5 | (7.2, 15.1) |  |  |
| 48-59 | - | - | - |  | 53 | 15.0 | (11.4, 19.5) |  |  |
| Sex |  |  |  |  |  |  |  |  |  |
| Male | 8 | 18.7 | (8.4, 36.5) | 0.701 | 95 | 11.8 | (9.5, 14.5) | 0.097 |  |
| Female | 11 | 16.0 | (7.5, 30.9) |  | 67 | 9.2 | (7.3, 11.5) |  |  |
| Residence |  |  |  |  |  |  |  |  |  |
| Rural | 8 | 19.3 | (7.4, 41.5) | 0.804 | 55 | 13.2 | (9.8, 17.5) | 0.208 |  |
| Urban | 9 | 15.2 | (6.4, 31.9) |  | 84 | 9.2 | (7.4, 11.3) |  |  |
| IDP | 2 | 25.0 | (3.8, 73.5) |  | 23 | 11.1 | (6.6, 17.9) |  |  |
| State |  |  |  |  |  |  |  |  |  |
| Somaliland | 4 | 9.0 | (3.6, 21.1) | 0.285 | 37 | 7.3 | (5.5, 9.8) | 0.006 |  |
| Puntland | 10 | 32.5 | (16.4, 54.2) |  | 44 | 15.6 | (11.7, 20.4) |  |  |
| Hirshabelle | 2 | 11.1 | (2.2, 41.5) |  | 25 | 13.2 | (7.6, 21.8) |  |  |
| South-West | - | - | - |  | 8 | 15.0 | (9.1, 23.8) |  |  |
| Jubaland | 2 | 50.0 | (1.8, 98.2) |  | 7 | 15.9 | (10.2, 24.0) |  |  |
| Banaadir | 1 | 21.4 | (3.4, 67.7) |  | 41 | 11.0 | (7.4, 16.0) |  |  |
| Wealth Quintile |  |  |  |  |  |  |  |  |  |
| Poorest | 0 | 0.0 | - | 0.790 | 9 | 11.7 | (6.3, 20.6) | 0.016 |  |
| Second | 2 | 21.3 | (4.3, 62.1) |  | 22 | 16.4 | (11.0, 23.8) |  |  |
| Middle | 6 | 21.9 | (8.3, 46.4) |  | 55 | 12.4 | (9.2, 16.6) |  |  |
| Fourth | 6 | 19.0 | (7.9, 39.2) |  | 43 | 9.5 | (6.9, 12.9) |  |  |
| Richest | 5 | 13.0 | (4.5, 32.3) |  | 31 | 6.8 | (4.7, 9.8) |  |  |
| Exclusive breastfeeding |  |  |  |  |  |  |  |  |  |
| No | 19 | 21.7 | (11.5 37.2) | 0.093 | - | - | - |  |  |
| Yes | 0 | 0.0 | -- |  | - | - | - |  |  |
| Ever breastfed |  |  |  |  |  |  |  |  |  |
| No | 2 | 17.1 | (3.5, 54.0) | 0.981 | - | - | - |  |  |
| Yes | 17 | 17.4 | (9.2, 30.4) |  | - | - | - |  |  |
| Diarrhea in past 2 weeks |  |  |  |  |  |  |  |  |  |
| No | 12 | 12.0 | (6.2, 21.9) | 0.001 | 141 | 10.9 | (9.2, 12.9) | 0.177 |  |
| Yes | 7 | 53.3 | (25.7, 79.1) |  | 21 | 8.0 | (5.0, 12.4) |  |  |
| LRI in past 2 weeks |  |  |  |  |  |  |  |  |  |
| No | 19 | 18.1 | (9.6, 31.5) | 0.363 | 143 | 10.1 | (8.5, 11.9) | 0.024 |  |
| Yes | 0 | 0.0 |  |  | 19 | 16.4 | (10.7, 24.3) |  |  |
| Inflammation |  |  |  |  |  |  |  |  |  |
| No | - | - | - |  | 90 | 9.8 | (7.8, 12.3) | 0.564 |  |
| Yes | - | - | - |  | 38 | 11.1 | (7.9, 15.5) |  |  |
| Iron status |  |  |  |  |  |  |  |  |  |
| Sufficient | - | - | - |  | 75 | 11.7 | (9.1, 15.0) | 0.088 |  |
| Deficient | - | - | - |  | 53 | 8.5 | (6.5, 11.0) |  |  |
| Vitamin A status |  |  |  |  |  |  |  |  |  |
| Sufficient | - | - | - |  | 86 | 10.1 | (7.9, 12.8) | 0.850 |  |
| Deficient | - | - | - |  | 42 | 10.4 | (8.0, 13.4) |  |  |
| Household sanitation ^f^ |  |  |  |  |  |  |  |  |  |
| Inadequate | 8 | 18.5 | (9.2, 33.6) | 0.861 | 84 | 10.4 | (8.2, 13.0) | 0.993 |  |
| Adequate | 11 | 16.6 | (5.8, 39.4) |  | 75 | 10.4 | (8.3, 13.0) |  |  |
| Safe drinking water |  |  |  |  |  |  |  |  |  |
| Unsafe | 6 | 22.8 | (8.3, 49.0) | 0.508 | 26 | 10.2 | (7.3, 13.9) | 0.864 |  |
| Safe | 13 | 15.9 | (7.6, 30.4) |  | 134 | 10.5 | (8.7, 12.6) |  |  |
| Any Soap |  |  |  |  |  |  |  |  |  |
| No | 4 | 16.6 | (4.9, 43.6) | 0.915 | 43 | 9.8 | (7.3, 13.2) | 0.622 |  |
| Yes | 15 | 17.5 | (10.2, 28.3) |  | 117 | 10.7 | (8.8, 13.0) |  |  |
| Household food security |  |  |  |  |  |  |  |  |  |
| Food secure | 5 | 10.3 | (3.3, 27.5) | 0.428 | 65 | 9.9 | (7.7, 12.7) | 0.914 |  |
| Mild insecurity | 1 | 20.3 | (2.5, 71.4) |  | 8 | 9.5 | (3.6, 22.9) |  |  |
| Moderate insecurity | 3 | 24.1 | (7.2, 56.5) |  | 18 | 10.2 | (6.2, 16.2) |  |  |
| Severe insecurity | 10 | 22.0 | (10.9, 39.4) |  | 69 | 11.1 | (8.6, 14.2) |  |  |
| Percent inadequate sanitation in cluster |  |  |  |  |  |  |  |  |  |
| 0-19% | 2 | 10.4 | (3.2, 29.0) | 0.174 | 23 | 11.9 | (8.2, 16.9) | 0.198 |  |
| 20-39% | 10 | 35.0 | (9.9, 72.4) |  | 42 | 12.4 | (8.5, 17.7) |  |  |
| 40-59% | 3 | 7.6 | (1.7, 28.7) |  | 36 | 8.8 | (6.1, 12.4) |  |  |
| 60-79% | 3 | 23.4 | (7.7, 52.9) |  | 19 | 7.2 | (4.4, 11.7) |  |  |
| 80-100% | 1 | 7.8 | (1.1, 38.2) |  | 40 | 12.4 | (9.3, 16.5) |  |  |
| **ALL CHILDREN** | **19** | **17.2** | **(9.2, 30.0)** |  | **162** | **10.5** | **(8.9, 12.4)** |  |  |
| Note: The n’s are un-weighted numerators for each subgroup; subgroups that do not sum to the total have missing data.  ^a^ Percentages weighted for unequal probability of selection  ^b^ CI=confidence interval, calculated taking into account the complex sampling design  ^c^ P-value <0.05 indicates that at least one subgroup is statistically significantly different from the other subgroups.  ^d^ Any stunting is defined as WHZ < -2 calculated using the WHO Child Growth Standards  ^f^ Composite variable of toilet type and if toilet facilities are shared with non-household members; Adequate Sanitation = flush or pour flush toilet or pit latrine with slab not shared with another household. Inadequate sanitation= open pit, bucket latrine, hanging toilet/latrine, no facility, bush, field | | | | | | | | | |
